# 

**Published:** 1974-04

**Authors:** 


Bristol Medico-Chirurgical Journal Vol. 89 (ii) No. 330

### Contents

Some Surgical Problems of the Newborn ... ... ... ... ... ... 11

A. G. McPherson

The Medical Library ... ... ... ... ... ... ... ... 16

The Management of Nasopharyngeal Angiofibroma ... ... ... ... 17
R. Kenneth Roddie

Bristol Medico-Chirurgical Society ... ... ... ... ... ... 22

News and Notes ... ... ... ... ... ... ... ... ... 22

